# Similar Pathogen Targets in *Arabidopsis thaliana* and *Homo sapiens* Protein Networks

**DOI:** 10.1371/journal.pone.0045154

**Published:** 2012-09-21

**Authors:** Paulo Shakarian, J. Kenneth Wickiser

**Affiliations:** 1 Paulo Shakarian Department of Electrical Engineering and Computer Science, United States Military Academy, West Point, New York, United States of America; 2 J. Kenneth Wickiser Department of Life Science, United States Military Academy, West Point, New York, United States of America; University of Georgia, United States of America

## Abstract

We study the behavior of pathogens on host protein networks for humans and *Arabidopsis* - noting striking similarities. Specifically, we preform 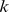-shell decomposition analysis on these networks - which groups the proteins into various “shells” based on network structure. We observe that shells with a higher average degree are more highly targeted (with a power-law relationship) and that highly targeted nodes lie in shells closer to the inner-core of the network. Additionally, we also note that the inner core of the network is significantly under-targeted. We show that these core proteins may have a role in intra-cellular communication and hypothesize that they are less attacked to ensure survival of the host. This may explain why certain high-degree proteins are not significantly attacked.

## Introduction

Recently, the work of Mukhtar et al. [1], [2] mapped protein interactions from the reference plant *Arabidopsis thaliana* (hereafter, *Arabidopsis*) and two pathogenic effectors. Additionally, the recent work of Navratil et al. [3] studied a human protein interaction network and its interactions with 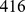 viral proteins. In this paper, we perform 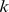-shell decomposition analysis [4]–[6] and other techniques on these networks. In doing so, we are able to identify several interesting aspects of the behavior of the pathogens with respect to both species. First, we observe a strong power-law correlation between the average degree of certain parts of the network called shells and the average number of pathogen interactions for each node. This provides us some insight on which parts of the network are more attacked by the pathogens. We also show that the proteins most often attacked tend to lie in the higher numbered shells (i.e. more toward the “core” of the network). Next, we find that the nature of the attack of the pathogens is somewhat limited in that an important structural component - the core - of both networks is significantly under-attacked. Finally, we also present some species-specific results for the two protein networks.

## Results

### Shells of Higher Average-Degree are Targeted by Pathogens

**Figure 1:**
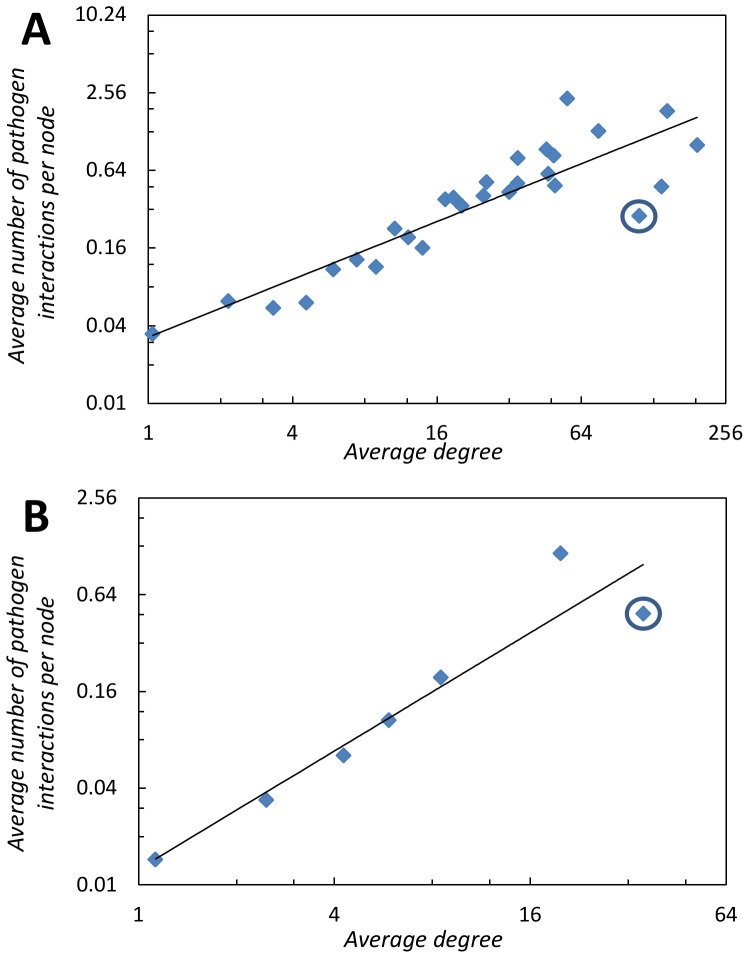
Average degree (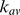) vs. average number pathogen interactions (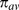) per node in a shell (log-log scale) with power-law fits. The core of each network is circled. (A) Human protein interaction network (B) *Arabidopsis* protein interaction network.

**Figure 2:**
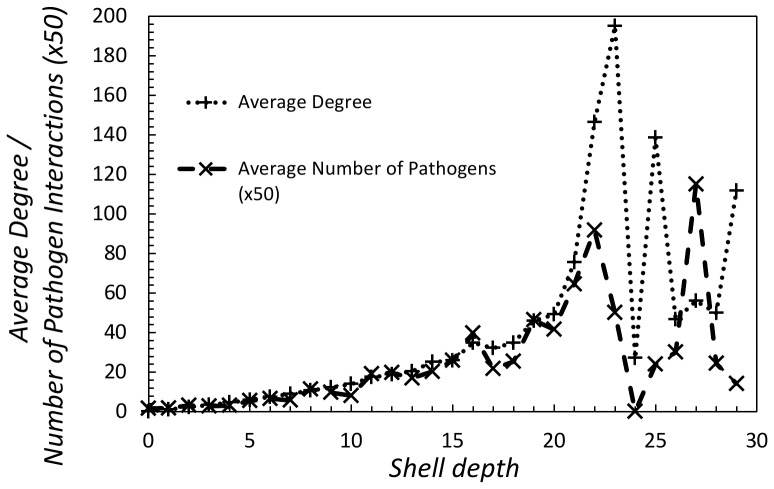
The average degree of each shell in the human protein interaction network increases for the first 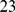 shells before becoming irregular. The average number of pathogen interactions for each shell follows a similar pattern.

Here we discuss that certain portions of the protein networks known as “shells” are more heavily targeted by the pathogens. The shells are determined using 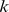-shell decomposition analysis. This procedure systematically divides the networks into sub-networks called shells (details on this procedure are in the Materials and Methods section). We show in Figure 1 a strong power-law correlation between the average degree (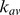) and average number of pathogen effectors per protein (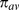) in each shell for both networks - despite being associated with species from different kingdoms (for humans, power-law regression produces 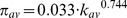, 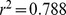, 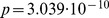, 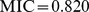; for *Arabidopsis*, 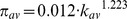, 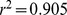, 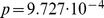, 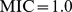). For cross-validation, we also measure correlation with the Maximal Information Coefficient [7] (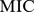), which does not assume a linear relationship (for details see the Materials and Methods section). Our finding suggests that pathogens seem to target proteins located in the more dense shells of the networks (shells with a higher average degree). Previous attempts to relate network measures with the behavior of the pathogens have provided only weak correlation (e.g. regression analysis correlating degree to number of pathogen interactions in the human protein networks gives 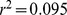 as reported by Navratil et al. - we provide a complete summary of these correlations later in the paper). With the *Arabidopsis*, Mukhtar et al. [1] shows that proteins of degree 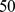 or greater were often interacted more with the pathogens, but do not show a correlation - most likely because many high degree proteins in that network were not significantly affected by pathogens. This study differs from these previous attempts in that we focus on layers of the networks as opposed to individual nodes. We also note that if, based on certain observable irregularities, we dis-regard a small handful of shells in the human protein network, that the correlation significantly increases. In the human protein network, the average degree monotonically increases with the first 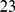 shells then becomes irregular. Further, the average number of pathogen interactions in each shell follows a very similar pattern - generally increasing during the first 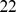 shells before becoming irregular (see Figure 2). Although we are unsure why this occurs, we do note that if power-law analysis is performed on the data for the first 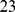 shells, the correlation significantly increases (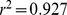, 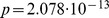, 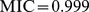, the 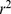 value when all shells are considered is 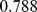, 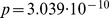, 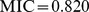).

**Figure 3:**
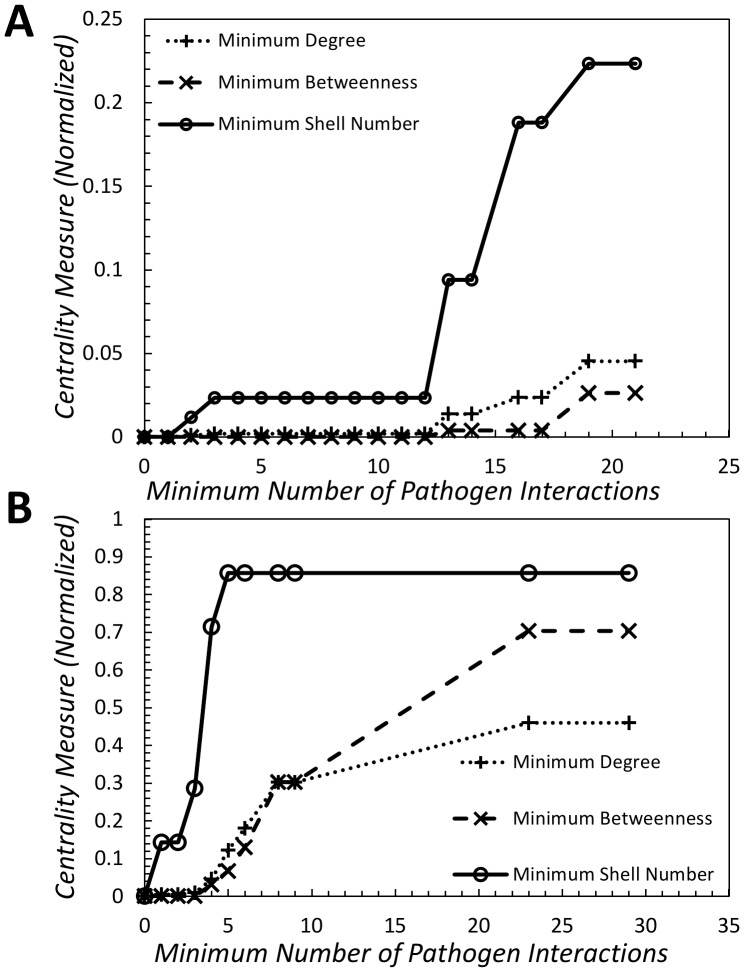
Normalized minimum centrality measure (the centrality measures depicted here are degree, betweenness, and shell number) of nodes targeted by at least a certain number of pathogen interactions. (A) Human protein interaction network. (B) *Arabidopsis* protein interaction network. Betweenness is defined in the Materials and Methods.

Can we extend the above analysis to identify highly-targeted proteins? Unfortunately, as with degree and betweenness (betweenness is defined in the Materials and Methods section), it appears that the shell number for each node is not correlated with number of pathogen effectors. However, can we extract a rule from the data of the form “if node 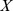 is targeted by at least 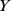 pathogen interactions then it must have a shell number of at least 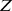
”? We looked at the minimum shell number targeted by a certain number of pathogen effectors (or greater) and found that such rules appear to be true for both datasets we examined. Figure 3 illustrates this relationship. We normalized the minimum shell-number, degree and betweenness associated with nodes being targeted by at least a certain number of pathogen interactions. It is noteworthy that the results for the human protein-interaction network, though striking, may actually be understated as the shell number (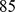) of the core is significantly higher than the next shell (which has a shell number of 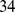).

### Core Network Proteins Under-Targeted

The core nodes of the network (the nodes in the inner-most shell) are consistently less attacked by the pathogens than expected. The core nodes (a.k.a. network nucleus), are a relatively small set of densely-connected nodes (a node in the core has many connections to other nodes in the core) that in other networks were shown to be associated with a key function of the network [5] and/or are amplifying the spread of a phenomenon [6]. An example of such a function could be the spread of information. Hence, targeting the core nodes does not seem to be part of the pathogen attack strategy in both humans and *Arabidopsis*. In the *Arabidopsis* network, core nodes are targeted by pathogens about half as expected based on the power-law correlation. For the human, these nodes are targeted only about a quarter as expected (the core nodes are circled in Figure 1). Further, it appears that highly targeted nodes are not found in the core. Of the top 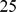 (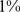) of targeted nodes in the *Arabidopsis* network, only one of them is in the core. Of the top 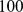 (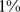) of targeted nodes in the human network, only two are in the core. In Mukhtar et al. [1] the authors examine nodes with a degree of 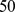 or greater - a set of 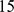 nodes referred to as 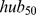 in the *Arabidopsis* network. Five of these are what those authors consider “highly targeted” and none of them are in the core. Of the remaining 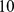 nodes in 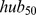, six of them are in the core. Membership in the core appears to correlate with high-degree nodes not being targeted.

There exists an optimal virulence - the degree of pathogenicity an infecting microbe has upon its host - that depends upon the fitness of both the host and the infecting entity [8]. The pathogen relies upon the host cellular machinery for replication so it impacts the genetic network involved in the immune response, pathogen infection process, and gene expression architecture to produce pathogen offspring. On the route to achieving optimal virulence, a pathogen may evolve to target the genetic circuits to allow maximum fitness of both itself and the host [9]. While some highly connected nodes of a genetic network prove to be lethal when either knocked down experimentally or perturbed by a pathogen in nature, the data analyzed herein demonstrates a plethora of viruses, bacteria, and eukaryotic pathogens target well-connected, but non-core nodes in both plant and human interactome datasets. It may be that the step-wise evolution of a pathogen involves the sampling of different host protein circuits in an effort to ensure optimum host viability and maximum pathogen replication [10], [11]. The targeting of high density, but non-core proteins may reflect an evolved strategic solution pathogens have employed to achieve optimal virulence [12]. The high density of the targeted nodes may accelerate a pathogen's ability to adapt to selective pressure by switching to a closely related target or it may simply reflect the highly connected nature of the targeted suite of proteins involved with the control of gene expression and cellular metabolism.

The intra-cellular communication circuits include signals transduction components between organelle such as the nucleus and mitochondria as the cell strives to maintain homeostasis. Many of these communication circuits are involved with host metabolism and are the same proteins co-opted to construct pathogen progeny. We now illustrate how nodes in and near the core can be viewed as superior spreaders of information by examining the information centrality [13]–[15] (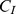) of the proteins in the various shells. Hence, nodes with high information centrality are thought to be excellent spreaders of information. Information centrality is more formally defined it the Materials and Methods section.

**Figure 4:**
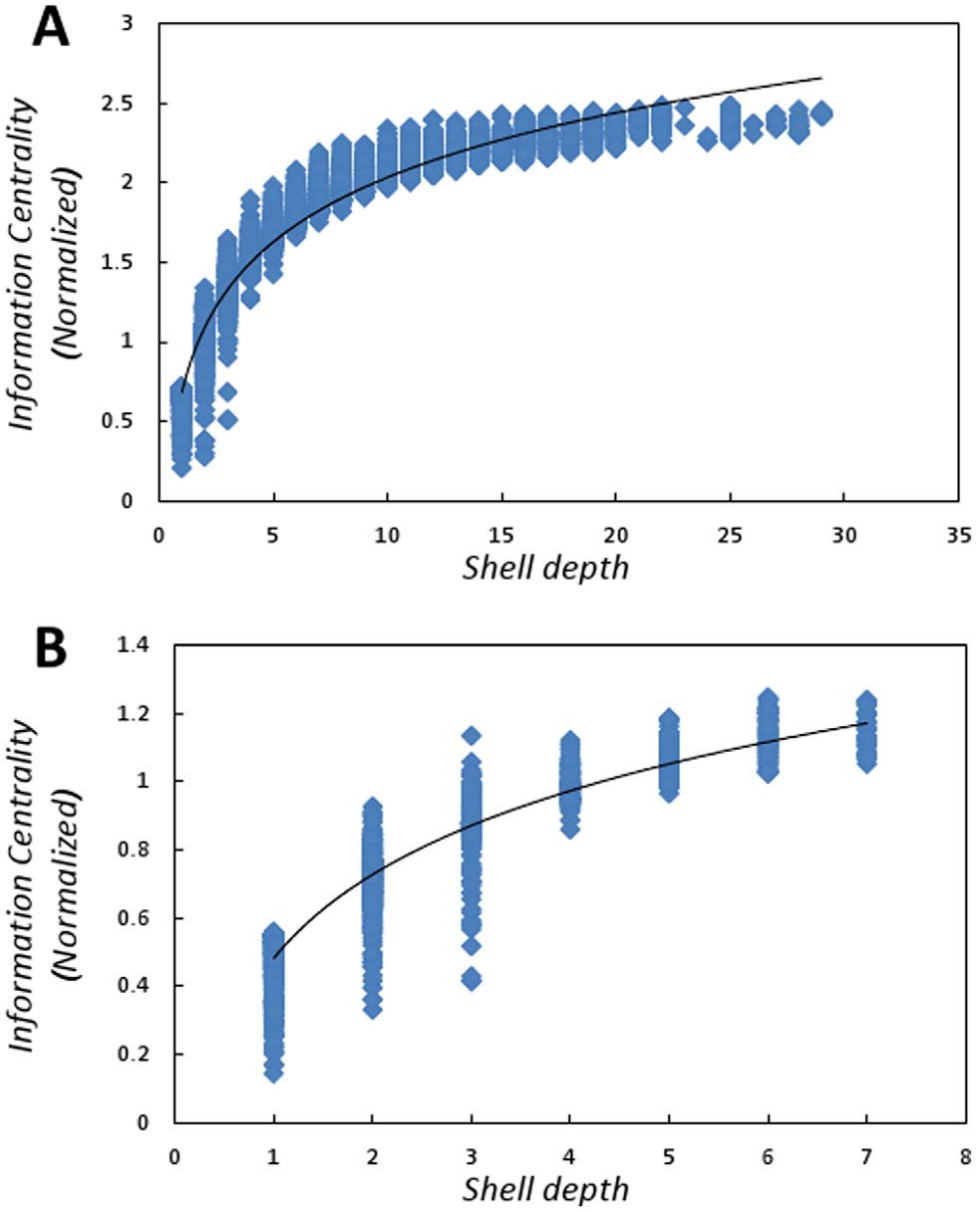
Shell depth vs. information centrality: (A) human protein interaction network, (B) *Arabidopsis* protein interaction network. Information centrality is defined in the Materials and Methods.

In both protein networks explored in this paper, the nodes in and near the core are superior spreaders of information given the information centrality of the proteins in various shells. We found a strong logarithmic correlation between shell depth (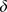) and information centrality in both the human and *Arabidopsis* protein networks (for humans, the relationship is 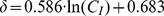, 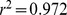, 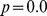, 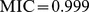; for *Arabidopsis*, the relationship is 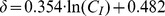, 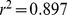, 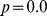, 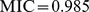, see Figure 4). In general, nodes toward the more inner shells have greater information centrality.

### Identifying Highly-Targeted Proteins in the *Arabidopsis* Network

**Figure 5:**
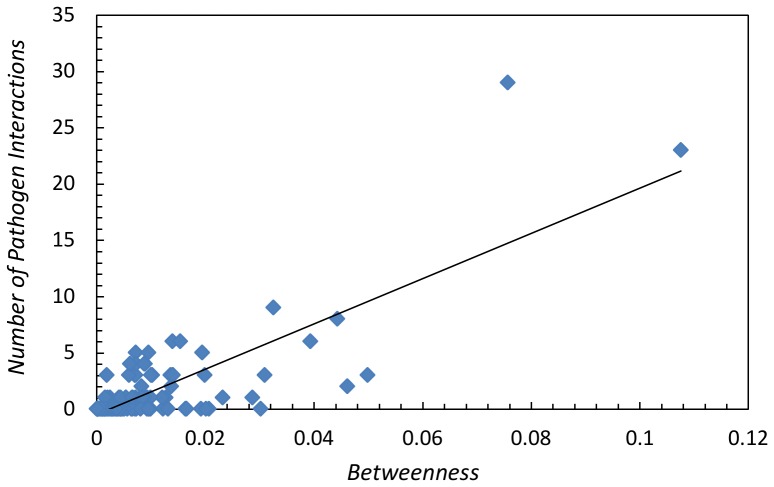
Betweenness centrality (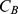) vs. number of pathogen interactions (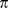) for the nodes in shell 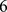 of the *Arabidopsis* protein interaction network.

Within a given shell of the *Arabidopsis* protein interaction network, the correlation coefficient (based on Pearson's 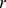, linear) for betweenness (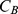) and number of pathogen interactions per node (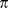) monotonically increased with the average degree of a given shell (power law regression fit yields 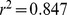, 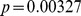, 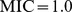,). This proves to be useful information in identifying high-targeted nodes as the shell with the highest average degree (shell 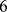, the last shell before the core, containing 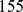 nodes) appeared to have a correlation for betweenness-number of pathogen interactions (linear regression fit gives 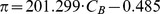, 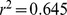, 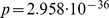, 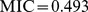). This is significantly greater than the linear-fit for the relationship among betweenness-number of pathogen interactions for the entire dataset (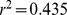, 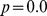, 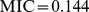). The relationship for this shell is shown graphically in the Figure 5. As an anecdote, the top 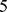 attacked proteins in the entire network were all contained within the top 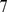 high-betweenness proteins of shell 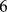. We note that similar results described in this paragraph can be derived based on degree-number of pathogen correlations. However, this is most likely a side-effect of the high correlation among degree and betweenness within shell 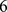 of the *Arabidopsis* protein interaction network (linear regression 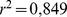, 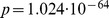, 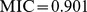).

### Characterizing Attacks on Essential Proteins in the Human Network

**Figure 6:**
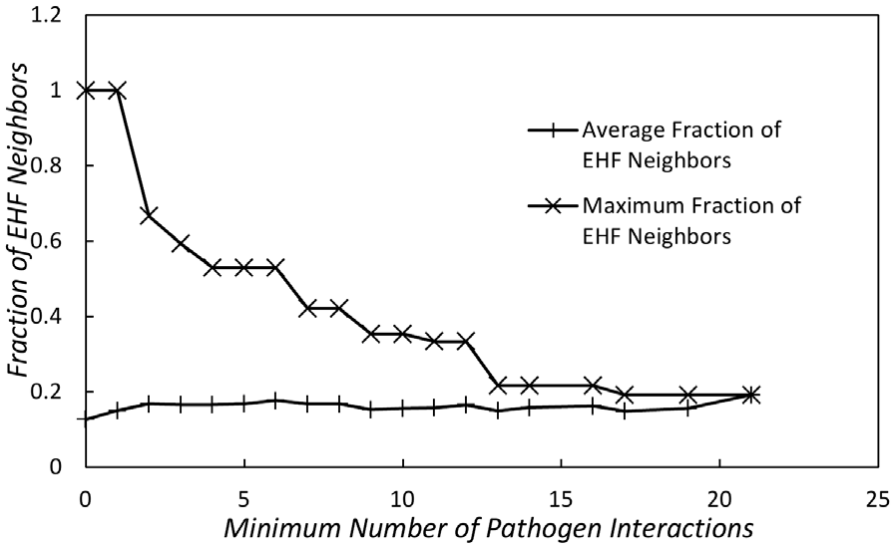
Minimum number of pathogen interactions for a given node vs. fraction of EHF neighbors for that node.

For the human network, we also further examined the relationship between essential host factors (EHF's) and the pathogen interactions. We used a list of 1501 EHF's from Navratil et al. [3]. In that work, the authors noted that over 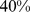 of the EHF's were within the local neighborhood (within 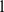 edge or less distance) from a targeted protein. While this indicated that pathogens target areas of the protein network near EHF's, it may be that the pathogens limit their attack to ensure the survival of the host (i.e. as with the lower-than-expected attachs of the core proteins we noted earlier). To examine this issue, we studied the average and maximum number of EHF proteins that are neighbors of node attacked by a certain number of pathogen effectors. We found that the average percentage of EHF neighbors remained at 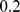 while the maximum decreased as the minimum number of pathogen interactions increased - see Figure 6.

### Notes on Essential Host Factors in the Human PIN

**Figure 7:**
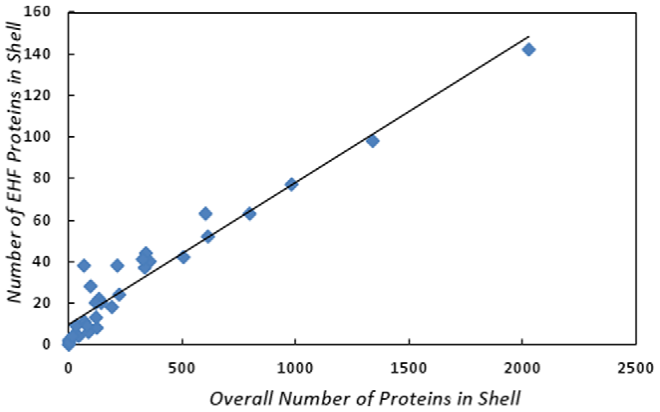
Total number of proteins vs. number of EHF proteins in a shell.

**Figure 8:**
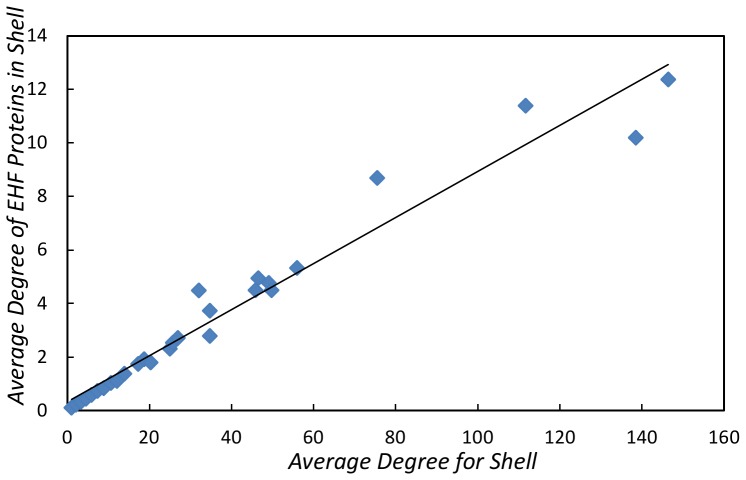
Average node degree vs. average EHF node degree in a shell.

We also found some results concerning the relationship between EHF proteins and network structure. In Figure 7, we show that there is a linear relationship between the size of a shell and the number of EHF proteins in that shell (where 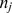 (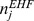) is the number of nodes (EHF nodes) in shell 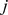 the relationship is 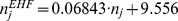, 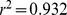, 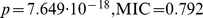). We also noticed (in Figure 8) that there is a linear relationship between the average degree of proteins in a shell and the average degree of EHF proteins in a shell (the relationship is 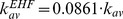, 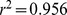, 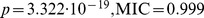).

### Correlation Studies On Node Centrality vs. Number of Pathogen Interactions Per Node (Negative Result)

We found a negative result on the correlation between node centrality measures and number of pathogen interactions per node. In general, there was little correlation found using linear regression. Similar results were obtained using power-law regression. On the human protein network, the 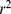 value associated with linear regression on degree-pathogen correlation is 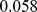 (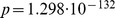, note that Navratil et al. report a slightly higher value - their analysis is most likely based on a power-law correlation) while 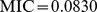, for betweenness-pathogen correlation is 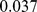 (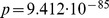) while 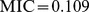, and for shell number-pathogen correlation is 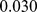 (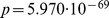) while 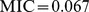. On the *Arabidopsis* protein network, the 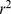 value associated with linear regression on degree-pathogen correlation is 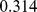 (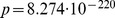) while 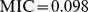, for betweenness-pathogen correlation is 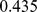 (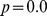) while 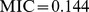, and for shell number-pathogen correlation is 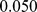 (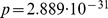) while 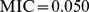.

## Discussion

We believe these results are exciting as they show that pathogen effectors seem to attack protein networks of entirely different organisms in very similar ways. Further, through 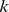-shell decomposition and regression analysis, we are able to identify high-risk shells for attack. We are currently looking to extend this work by creating software tools to extract highly-relevant patterns of pathogens attacks. Other future work of interest would be to explore pathogen relationships with host protein networks for other organisms.

## Materials and Methods

The degree of a host protein node in the networks considered is the number of other host proteins interacting with it. The number of interacting pathogens for a given protein node (denoted by 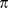 in this paper) is the total number of proteins in all pathogen protein networks (considered for that host species) which interact with that protein node.

The 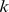-shell decomposition method can be described as follows. At the first iteration, all unconnected nodes are removed and are considered to be in shell 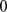 (note that we did not consider this shell in our analysis – as it has been observed that unconnected proteins were largely unaffected by pathogens [1], [3]). Then all nodes connected to the graph by one edge are removed, they are in shell 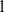. Upon their removal, there may be other nodes connected to the graph by one edge or less - they too are removed and are also considered in shell number 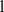 (we then continue removing nodes from the graph for shell 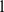 until there are no more nodes connected by just one edge or less). The process repeats for nodes connected to the graph with only two edges (they are in shell 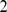) and so on until nodes are removed. The nodes in the highest 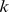-shell are known as the core. We define the term “shell depth” as the number representing the order in which the shell is determined - for example if shell number 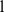 is followed by shell number 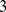, then the depth of shell 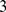 would be 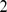. We define “shell size” simply as the number of nodes in a particular shell.

Betweenness centrality, [16], often simply called “betweenness,” is defined as follows. Let 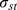 be the number of shortest paths between nodes 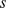 and 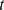 and 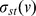 be the number of shortest paths between 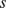 and 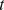 containing node 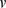. Betweenness centrality for node 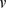 is then 
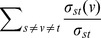
. Intuitively, nodes of high-betweenness can be thought of as “bottlenecks” as their removal often results in an increase in shortest path length between node pairs in the network. The NetworkX package used in our analysis implements the algorithm of [17] to compute this measure.

Information centrality [13], studies all different paths between two nodes in a network. In [13], the information value between two nodes is related to the inverse length of the different paths between them. For node pair 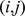, they define a square matrix 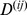 where the number of rows in the matrix is equal to the total number of paths between 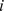 and 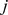. The 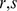 component of the matrix, 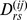, is equal to the number of shared links between the path specified at row 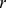 and row 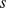. Hence, for undirected networks (as the protein networks used in this paper) the matrix is symmetric. To define the information between 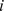 and 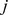, denoted 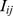, the authors sum the components of the inverse of 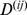. Based on this calculation, for a network of 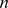 nodes, the information centrality of a given node 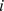 (denoted 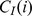) is defined as follows.
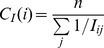
(1)Hence, the information centrality of node 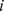 is the harmonic mean of the information associated with the paths from 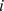 to all other nodes in the network.

The *Arabidopsis* protein network of Mukhtar et al. [1] consisted of 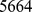 interactions among 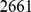 proteins and 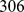 interactions with pathogens. The maximum degree was 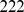 and excluding unconnected nodes the network was decomposed into 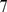 shells. The human protein interaction network of Navratil et al. [3] consisted of 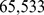 interactions among 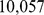 proteins. There were 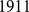 interactions with pathogens. The maximum degree was 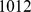. Decomposed, the human network had 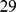 shells.

All network analysis was performed using NetworkX (http://networkx.lanl.gov/) and all statistics were performed using SciPy (http://www.scipy.org/).

In the power-law regression analysis of the human network, shell 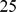 (consisting of three nodes with degrees 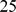, 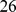, and 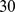) was omitted from the power-law analysis as it was not affected by any virus. For the high-quality human network, shell 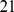 (consisting of one node with a degree of 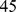) was omitted for the same reason. No shells were omitted in analysis done with the Maximum Information Coefficient.

For all linear regression analysis, the 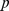-value (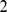-tailed unless specified otherwise) refers to the probability that the slope is zero (roughly the probability that an uncorrelated system produces datasets that have an 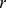 value greater than or equal to one reported). For power regression, this refers to the probability that the scaling exponent is zero.

In addition to the normal regression analysis, we also computed the Maximal Information Coefficient (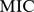) [7] that measures the correlation of two variables without assuming a linear relationship. This coefficient is a number in the interval 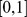 that monotonically increases with correlation. We used the the MINE software available from http://exploredata.net to compute this quantity. Note that if we compared two variables, the 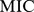 was computed on the two original variables (i.e. not the logarithm).

For the results on information centrality, all of our results are on the greatest connected components of either graph. This is because information centrality [13]–[15] is only defined for strongly connected graphs.

For our EHF results, the set of “EHF neighbors” includes all the neighbor of a given node and itself. Hence, we assume a self-loop. For instance, an EHF protein not adjacent to any other EHF protein has one “neighbor” – itself.

## References

[1] Mukhtar MS, Carvunis AR, Dreze M, Epple P, Steinbrenner J, et al (2011) Independently Evolved Virulence Effectors Converge onto Hubs in a Plant Immune System Network. Science 333: 596–601. 21798943 10.1126/science.1203659 PMC3170753

[2] Consortium AIM (2011) Evidence for Network Evolution in an Arabidopsis Interactome Map. Science 333: 601–607. 21798944 10.1126/science.1203877 PMC3170756

[3] Navratil V, de Chassey B, Combe CRR, Lotteau V (2011) When the human viral infectome and diseasome networks collide: towards a systems biology platform for the aetiology of human diseases. BMC systems biology 5: 13+. 21255393 10.1186/1752-0509-5-13 PMC3037315

[4] Seidman S (1983) Network structure and minimum degree. Social Networks 5: 269–287.

[5] Carmi S, Havlin S, Kirkpatrick S, Shavitt Y, Shir E (2007) From the Cover: A model of Internet topology using k-shell decomposition. PNAS 104: 11150–11154. 17586683 10.1073/pnas.0701175104 PMC1896135

[6] Kitsak M, Gallos LK, Havlin S, Liljeros F, Muchnik L, et al (2010) Identification of inuential spreaders in complex networks. Nat Phys 6: 888–893.

[7] Reshef D, Reshef Y, Finucane H, Grossman S, McVean G, et al (2011) Detecting novel associations in large data sets. Science 334. 10.1126/science.1205438 PMC3325791 22174245

[8] Jensen K, Little TJ, Skorping A, Ebert D (2006) Empirical support for optimal virulence in a castrating parasite. PLoS Biology 4. 10.1371/journal.pbio.0040197 PMC1470460 16719563

[9] Berenos C, Schmid-Hempel P, Wegner K (2011) Experimental coevolution leads to a decrease in parasite-induced host mortality. Journal of Evolutionary Biology 24. 10.1111/j.1420-9101.2011.02306.x 21599776

[10] Smith J (2007) A gene's-eye view of symbiont transmission. American Naturalist 170: 542–550. 10.1086/521236 17891733

[11] Kover P, Clay K (1998) Trade-off between virulence and vertical transmission and the maintenance of a virulent plant pathogen. American Naturalist 152: 165. 10.1086/286159 18811383

[12] Best A, White A, Boots M (2009) The implications of coevolutionary dynamics to host-parasite interactions. American Naturalist 173: 779. 10.1086/598494 19374557

[13] Stephenson K, Zelen M (1989) Rethinking centrality: Methods and examples. Social Networks 11: 1–37.

[14] Noh JD, Rieger H (2004) Random walks on complex networks. Physical Review Letters 92: 118701. 15089179 10.1103/PhysRevLett.92.118701

[15] Brandes U, Fleischer D (2005) Centrality measures based on current ow. In: STACS. pp. 533–544.

[16] Freeman LC (1977) A set of measures of centrality based on betweenness. Sociometry 40: 35–41.

[17] Brandes U (2001) A faster algorithm for betweenness centrality. Journal of Mathematical Sociology 25.

